# Evaluation of alternative mosquito sampling methods for malaria vectors in Lowland South - East Zambia

**DOI:** 10.1186/1756-3305-6-91

**Published:** 2013-04-09

**Authors:** Chadwick H Sikaala, Gerry F Killeen, Javan Chanda, Dingani Chinula, John M Miller, Tanya L Russell, Aklilu Seyoum

**Affiliations:** 1 National Malaria Control Centre, Chainama Hospital College Grounds, Off Great East road, P.O. Box 32509, Lusaka, Zambia; 2 Vector Biology Department, Liverpool School of Tropical Medicine, Pembroke Place, Liverpool, L3 5QA, UK; 3 Ifakara Health Institute, Biomedical & Environmental Thematic Group, P.O. Box 53, Ifakara, Morogoro, United Republic of Tanzania; 4 Malaria Control, Evaluation and Partnership in Africa (MACEPA), Chainama Hospital College Grounds, Off Great East road, P.O. Box 32509, Lusaka, Zambia; 5 James Cook University, School of Public Health, Tropical Medicine and Rehabilitation Sciences, Cairns, 4870, Australia

**Keywords:** *Anopheles quadriannulatus*, *Anopheles funestus*, *Sampling*, *Ifakara Tent Trap*, *Sensitivity*

## Abstract

**Background:**

Sampling malaria vectors and measuring their biting density is of paramount importance for entomological surveys of malaria transmission. Human landing catch (HLC) has been traditionally regarded as a gold standard method for surveying human exposure to mosquito bites. However, due to the risk of human participant exposure to mosquito-borne parasites and viruses, a variety of alternative, exposure-free trapping methods were compared in lowland, south-east Zambia.

**Methods:**

Centres for Disease Control and Prevention miniature light trap (CDC-LT), Ifakara Tent Trap model C (ITT-C), resting boxes (RB) and window exit traps (WET) were all compared with HLC using a 3 × 3 Latin Squares design replicated in 4 blocks of 3 houses with long lasting insecticidal nets, half of which were also sprayed with a residual deltamethrin formulation, which was repeated for 10 rounds of 3 nights of rotation each during both the dry and wet seasons.

**Results:**

The mean catches of HLC indoor, HLC outdoor, CDC-LT, ITT-C, WET, RB indoor and RB outdoor, were 1.687, 1.004, 3.267, 0.088, 0.004, 0.000 and 0.008 for *Anopheles quadriannulatus* Theobald respectively, and 7.287, 6.784, 10.958, 5.875, 0.296, 0.158 and 0.458, for *An. funestus* Giles, respectively. Indoor CDC-LT was more efficient in sampling *An. quadriannulatus* and *An. funestus* than HLC indoor (Relative rate [95% Confidence Interval] = 1.873 [1.653, 2.122] and 1.532 [1.441, 1.628], respectively, *P* < 0.001 for both). ITT-C was the only other alternative which had comparable sensitivity (RR = 0.821 [0.765, 0.881], *P* < 0.001), relative to HLC indoor other than CDC-LT for sampling *An. funestus*.

**Conclusions:**

While the two most sensitive exposure-free techniques primarily capture host-seeking mosquitoes, both have substantial disadvantages for routine community-based surveillance applications: the CDC-LT requires regular recharging of batteries while the bulkiness of ITT-C makes it difficult to move between sampling locations. RB placed indoors or outdoors and WET had consistently poor sensitivity so it may be useful to evaluate additional alternative methods, such as pyrethrum spray catches and back packer aspirators, for catching resting mosquitoes.

## Background

In measuring malaria transmission intensity under varying epidemiological settings, entomological sampling methods that catch mosquitoes with high sensitivity are very useful, particularly as vector densities drop in response to increasingly effective vector control and elimination of transmission is prioritised by an increasing number of countries [1-5]. Generally, these sampling methods involve collection of adult mosquitoes either indoors or outdoors, with the host-seeking females that mediate transmission as the primary target for trapping [5,6]. Human landing catch (HLC) is the gold standard method for collection of host-seeking mosquitoes [7] to determine their biting rate, infection prevalence, and consequently the intensity of malaria transmission they mediate. However, HLC raises ethical concerns because catchers are exposed to vectors that could be potentially infective. It is also labour intensive and unreliable due to variation in attractiveness and skill of the catchers who act as bait hosts [1,8-10]. The continued application of this tool in the surveillance of malaria transmission in sub-Sahara Africa requires careful re-examination and re-justification, with a view to developing and characterizing safer alternative tools that are comparably sensitive.

Over the years, a number of alternative sampling tools that avoid human contact with mosquitoes have been evaluated. These have exhibited wide variations in efficacy and cost, and may not be practical for adoption on programmatic scales in poor malaria-endemic countries [1,6]. One of the most commonly employed tools for catching host-seeking malaria vectors in particular is the Centres for Disease Control and Prevention miniature light trap (CDC-LT), which is typically positioned indoors near an occupied net [11,12]. Numerous studies have shown the effectiveness of CDC-LTs over a wide range of transmission systems in Africa [12-17]. The positioning of the CDC-LT during sampling influences the sensitivity with which it samples adult female mosquitoes [16] and this trap is almost equally effective when occupants are sleeping under a treated or untreated bed net [18,19].

However, where indoor-targeted insecticidal based interventions such as long-lasting insecticide treated nets (LLINs) and indoor residual spraying (IRS) have drastically reduced endophilic and endophagic vectors [20,21], traps for capturing host-seeking mosquitoes outside of houses are considered more suitable to sample the exophagic vectors that become increasingly important contributors to the residual vector population as intervention coverage is scaled up [4,22-25]. While capture methods primarily targeting host-seeking mosquitoes are ideal for quantifying human exposure to bites and studying host attack behaviours, resting and exit traps are more appropriate for studying resting behaviours and sampling fed mosquitoes to determine the source of blood obtained [5,6].

The characteristic indoor resting (endophilic) behaviour of *Anopheles gambiae* Giles, *An. arabiensis* Patton and *An. funestus* Giles underpins the common use of indoor knockdown pyrethrum spray catches (PSC) and hand collections using a mouth aspirator when surveying [6]. The major drawbacks associated with the hand collection method for resting mosquitoes is poor sensitivity, the laborious nature of rigorous searches through all the irregular surfaces of rural houses, and the great variability in skills and motivation among collectors [5,26]. PSC may be expensive to sustain for routine monitoring [26] while the repellence and persistence of the pyrethrum used precludes sampling in the same dwelling more than twice a week [5,27]. Other sampling methods such as, resting boxes (RB), clay pots and bed net traps have been evaluated under different epidemiological settings in Africa with varying degrees of success [28-37]. While window exit traps (WET) have been used for monitoring vector density trends in parts of southern Africa and Bioko island in central Africa [38,39], their efficacy is undoubtedly affected by variations in house design and behavioural patterns of both mosquitoes and humans [40].

A recent review [1] has highlighted the lack of consistency, comparability and characterisation of the numerous, diverse entomological survey tools used to measure malaria transmission. Recent evaluations of a newly developed Ifakara Tent Trap Design C (ITT-C) [41] show that, unlike the B design that preceded it [37,42], the ITT-C is a genuinely exposure-free tool that probably represents a relatively sensitive and practical mode of sampling malaria vectors for routine surveillance purposes [40], notably through community-based trapping schemes with epidemiological predictive power [43]. Here we report a comparative evaluation of the ITT-C, CDC-LT, RB and WET methods that do not necessitate increased human exposure to mosquito bites, compared to the gold standard HLC which does, in a rural part of Zambia with stable endemic transmission mediated primarily by *Anopheles funestus* Giles [44,45]. Insecticidal interventions, such as LLINs and IRS can alter survival rates, as well as entry, feeding, resting and exiting behaviours within houses [46], and these two interventions are sometimes combined in parts of Zambia and elsewhere in Africa, with the intention of achieving greater impact than with either alone [47-49]. The influence of supplementing LLINs with IRS upon the efficacy of these trapping methods was, therefore, also assessed by comparing capture rates and sample composition in and immediately outside of houses with both interventions versus those with LLINs alone.

## Methods

### Study area

**Figure 1:**
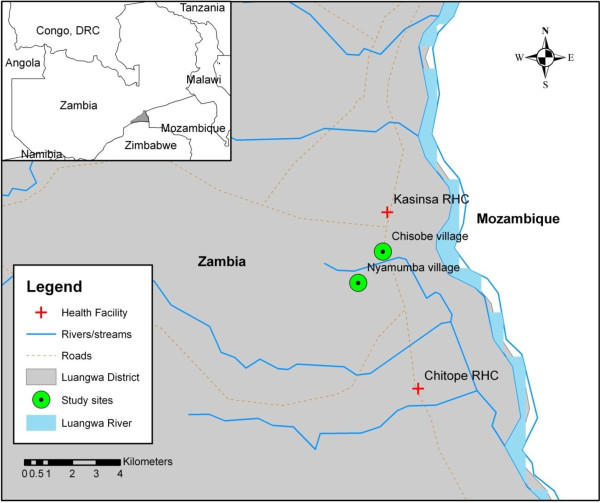
Location of study site (Chisobe and Nyamumba) in Luangwa district.

The study was conducted in Chisobe and Nyamumba villages situated between Kasinsa and Chitope rural health centres in Luangwa district (Figure 1) which is about 255 km east of Lusaka. Chisobe and Nyamumba are about 2 – 3 kilometres apart. Luangwa is at latitude −15°41’ E, and longitude 30°08’ S. It is approximately 370 m above sea level. The wet season runs from November to April and the dry season from June to September with October and May being transitional months. Annual rainfall varies from 600 to 1,400 mm with mean daytime temperatures ranging from 10°C to 44°C. There are about 26,000 inhabitants in the district who predominantly practice fishing. They also practice animal husbandry and grow seasonal crops.

### Study design

The study was conducted during two intervals chosen within the dry and wet seasons, specifically from September to October 2009 and from February to March 2010, respectively. A 3 × 3 Latin Square design was used for the rotated assignment of mosquito sampling methods to experimental units (houses). In each village (Chisobe and Nyamumba), two groups of three houses which were clustered together and identified as distinct experimental blocks with one group comprising those containing LLINs whilst the other also had LLINs but were also treated with IRS. IRS was applied using a deltamethrin formulation (K-Othrine® WG 250, Bayer Environmental Sciences) at a rate of 20 mg of active ingredient per m^2^ by an experienced spray operator trained at the National Malaria Control Centre.

At the time of the experiment, the only major intervention in the district was the use of PermaNet 2.0® LLINs (Vestergaard Frandsen SA) distributed through mass distribution campaigns and ante-natal clinics by the Ministry of Health and its partners. As IRS was not an intervention implemented in the district at that time, we therefore purposely sprayed only the selected houses in the LLINs plus IRS blocks to conform to the study design.

Each block was treated as a self-contained trio of numbered (1, 2 and 3) houses in which a Latin Squares rotation sequence was followed throughout the study period. In each of the blocks, the first treatment comprised the HLC conducted both indoors and outdoors and was randomly assigned to one of the numbered houses. The second treatment consisted of a CDC light trap beside an occupied LLIN inside the house, with an ITT-C (Elastic Products Manufacturing Co. Ltd, 67 Bibi Titi Mohamed Road, P.O. Box: 20872, Dar es Salaam, United Republic of Tanzania) placed approximately 5 metres outside of the house, and was assigned to the next highest number. The ITT-C is a canvas tent trap which is about 2000 mm long, 1000 mm wide and 1250 mm high with six funnel-shaped mosquito entrances which enables entry while restricting mosquito exit [41]. Two netting compartments are 700 mm apart and have sealable cotton sleeves to enable the collection of mosquitoes while avoiding bites. The collecting chambers are further supported with two strings to avoid collapse and further human-mosquito contact. The third treatment consisted of two resting boxes (one placed indoors and the other outdoor) and a window exit trap and was assigned to the next highest number. These collection methods have been described in detail in a similar study conducted in urban Tanzania [40]. Each of the three sets of indoor and outdoor collection methods was rotated through the three different houses in increasing order according to their assigned house number, for three consecutive nights in each of 10 rounds, to achieve a balanced data set reflecting an equal number of samples from each treatment-house combination, and time period (rounds). This series of 10 rounds of Latin Squares rotations in 4 blocks over a period of 30 consecutive nights was conducted in both the dry and wet seasons. To compensate for the relative attractiveness of individuals to mosquitoes [8,9] as a confounding factor, the same individual volunteers, who were retained in each house for the duration of the study, were exchanged between indoor (HLC or CDC-LT) and outdoor (HLC or ITT-C) stations each night in a crossover design. For the third treatment, where no human-baited outdoor catches were conducted, both volunteers slept within the house if they were from the same household, otherwise only the volunteer who owned the house and who subsequently conducted HLC indoors and slept under an LLIN when applying CDC-LT occupied the house. In order to ensure comparability, all methods for trapping host-seeking mosquitoes were conducted from 19:00 hrs to 07:00 hrs and all the RBs and WET were emptied at 07:00 hrs after operating for 12 hrs using hand held aspirators as described by Sikulu *et al*. (2009). Collections from the hourly catches from each catcher conducting HLC were placed in separate cups. Individuals collecting mosquitoes by HLC were allowed to rest for 15 minutes in each hour of collection. Approximately 20 minutes were required to aspirate mosquitoes from each of the ITT-C, CDC-LT, RBs and the WET methods. A team of supervisors conducted random and regular on spot checks to ensure that acceptable standards of execution were maintained by the volunteers.

### Mosquito processing

Mosquitoes were collected from each trap and identified in the field. Female *Anopheles* mosquitoes were identified to species morphologically [50] and preserved individually in silica gel. Male anophelines were only identified, recorded and discarded. They did not form any part of the analysis. *An. gambiae sensu lato* and *An. funestus sensu lato* samples were preserved for circumsporozoite ELISA for infectivity rates [51] and Polymerase Chain Reaction (PCR) for species identification [52,53]. Approximately 83% (1387) and 11% (932) of the total specimen morphologically identified as *An. gambiae s.l*. and *An. funestus s.l.,* respectively were analysed to determine species identity by PCR [52,53] and those which successfully amplified were used for further circumsporozoite ELISA analysis [51]. All identified culicine mosquitoes were recorded as either male or female and discarded.

### Data analysis

All the data were entered using the 2007 Microsoft Excel version. Analysis was performed following the Generalized Linear Mixed Models (GLMM) using R software version 2.15.1. augmented with the Matrix, lattice and lme4 packages. Mixed effects models were used so that fixed effects variables could be used to estimate the effect of factors of interest while accounting for repeated measurements and the influence of other variables such as date and household with many levels as random effects.

### Relative abundance, mean catches and sensitivity per sampling method

The relative catches of the female *An. quadriannulatus, An. funestus,* and other anopheline and culicine mosquitoes by the different mosquito sampling methods, as compared to the reference method (HLC-indoor), were analysed by fitting GLMMs as follows. The number of catches of the specific mosquito taxon was treated as the dependent variable, to which a Poisson distribution with a logarithm link function was applied. The sampling method, village, treatment (LLINs alone versus LLINs plus IRS) and season were fitted as fixed effects while date (d.f. = 60) and household (d.f. =12) were treated as random effects. The exponential of the parameter estimates (and 95% confidence intervals) for each method was calculated to represent the relative rate of catching mosquitoes compared to the standard reference method (HLC indoor). We calculated the mean by fitting GLMM with the sampling method treated as a categorical factor and both date and house as random effects using a Poisson distribution with logarithm link function and determined as described above. Similarly, we used the outputs from GLMM model to test for and quantify the effect of treatment, season and village on the abundance of mosquitoes of different taxa.

### Influence of indoor residual spraying upon the numbers of human-feeding *An. funestus* caught by all sampling methods

In order to analyse the effect of treating a house with IRS upon house entry and feeding on humans by mosquitoes, we fitted GLMMs with Poisson distribution, treating the number of mosquitoes caught with each trapping method in each house and station (in versus out) as the dependent variable and IRS treatment status, village and season as fixed effects. Household (d.f. =12) and date (d.f. =60) were treated as random effects.

### Influence of sampling method on the proportion of all *An. quadriannulatus* and *An. funestus* caught which were fed

In order to analyse the effect of trapping method upon the proportion of mosquitoes which had fed, we applied binomial logistic regression by fitting a GLMM with a logit link function for the proportion of fed female mosquitoes caught by each method, defined by the total number of fed mosquitoes as the numerator and the total catch of all female mosquitoes of all physiological status as the denominator. Abdominal status was classified as either fed (partly fed and fed) or unfed (unfed, partly gravid and fully gravid) and the fixed effects included village, season, and IRS treatment status while date and household were included as random effects.

### Ethical considerations

The study protocol was granted approval by The National Ethics Committee based at The University of Zambia (IRB00001131 of I0RG0000774) and the Ethical Review Board of the Liverpool School of Tropical Medicine (09.60). All individuals in the study had consented to participate in the study following a thorough description of the benefits and risks involved. Consenting participants were administered with Deltaprim^©^ drug (one of the recommended drugs for chemoprophylaxis in Zambia) as prophylaxis every week.

## Results

### Mean catches and relative sensitivities of alternative sampling methods in relation to indoor

### HLC

Summary of total catches, mean catches per trap night and relative sensitivities of alternative sampling methods in relation to HLC indoor are indicated in Table 1. A total of 19664 female mosquitoes were caught in 60 sampling nights, with 7.4% comprising *An. gambiae s.l.,* 38.9% *An. funestus s.l.,* 22.6% other anophelines and 31.1% culicine mosquitoes. The other anophelines constituted mainly *An. coustani, An. pretoriensis, An. squamosus* and *An. rufipes.* Out of the 932 (11%) specimens of *An. funestus s.l.*, tested by PCR, only 47% (n = 440) successfully amplified. Most were identified as *An. funestus sensu stricto* (72.2%, n = 317) with the remainder being *An. rivulorum* (16.2%, n = 71), *An. parensis* (9.8%, n = 43)*, An*. *vaneedeni* (1.4%, n = 7) and *An. lessoni* (0.5%, n = 2). From the total of 1387 (83%) *An. gambiae s.l.* specimens tested by PCR, 1169 (85%) successfully-amplified. The vast majority were *An. quadriannulatus* (95.2%, n = 1112) with only a very small number of *An. arabiensis* (3.9%, n = 46) and *An. gambiae sensu stricto* (0.9%, n = 11). In subsequent analysis, we therefore report results for the *Anopheles funestus* group and the *An. gambiae* complex as approximately representing *Anopheles funestus s.s. and An. quadriannulatus*, respectively*. Anopheles rivulorum* (18.3%, n = 13) and *An. funestus s.s* (2.2%, n = 7) were the only species from the *An. funestus* group, or any other *Anopheles* taxon, found to be infected with *P. falciparum* sporozoites. However, none of these specimens were re-tested following heating of the homogenates, so the possibility of exaggerated sporozoite prevalence due to false positives, for *An. rivulorum* in particular, cannot be excluded [54].

**Table 1:** **Number of mosquitoes caught by different sampling methods for 240 trap nights each and their relative rates in reference to the indoor human landing catches, as determined by fitting generalized linear mixed models**^**a**^

**Sampling method**	**Catch**^**b**^		**Relative sensitivity **^**c**^	
	**Total**	**Mean [95% CI]**	**RR [95% CI]**	***P *value**
*Anopheles quadriannulatus*				
HLC indoor	405	1.687 [1.531, 1.860]	1.00^d^	NA^e^
HLC outdoor	242	1.004 [0.885, 1.139]	0.597 [0.509, 0.700]	< 0.001
CDC light trap	784	3.267 [3.046, 3.504]	1.873 [1.653, 2.122]	0.997
Ifakara tent trap – C	21	0.088 [0.057, 0.134]	0.050 [0.032, 0.078]	< 0.001
Window exit trap	1	0.004 [0.001, 0.030]	0.002 [0.000, 0.015]	< 0.001
Resting boxes indoor	0	NE^f^	NE^f^	NE^f^
Resting boxes outdoor	2	0.008 [0.002, 0.033]	0.004 [0.001, 0.016]	< 0.001
*Anopheles funestus*				
HLC indoor	1749	7.287 [6.954, 7.637]	1.00^d^	NA^e^
HLC outdoor	1635	6.784 [6.463, 7.121]	0.928 [0.868, 0.993]	< 0.001
CDC light trap	2630	10.958 [10.547, 11.385]	1.532 [1.441, 1.628]	< 0.001
Ifakara tent trap – C	1410	5.875 [5.576, 6.190]	0.821 [0.765, 0.881]	< 0.001
Window exit trap	71	0.296 [0.234, 0.373]	0.040 [0.032, 0.051]	< 0.001
Resting boxes indoor	38	0.158 [0.115, 0.218]	0.022 [0.016, 0.030]	< 0.001
Resting boxes outdoor	110	0.458 [0.380, 0.553]	0.063 [0.052, 0.076]	< 0.001
Other anophelines				
HLC indoor	1661	8.046 [7.695, 8.413]	1.00^d^	NA^e^
HLC outdoor	2064	9.685 [9.300, 10.086]	1.207 [1.137, 1.287]	< 0.001
CDC light trap	661	2.754 [2.552, 2.972]	0.337 [0.308, 0.369]	< 0.001
Ifakara tent trap – C	28	0.117 [0.081, 0.169]	0.014 [0.010, 0.021]	< 0.001
Window exit trap	7	0.029 [0.014, 0.061]	0.003 [0.002, 0.007]	< 0.001
Resting boxes indoor	4	0.017 [0.006, 0.044]	0.002 [0.001, 0.005]	< 0.001
Resting boxes outdoor	20	0.083 [0.054, 0.129]	0.010 [0.006, 0.015]	< 0.001
Culicine species				
HLC indoor	1971	8.296 [7.939, 8.668]	1.00^d^	NA^e^
HLC outdoor	1921	8.033 [7.683, 8.399]	0.971 [0.912, 1.0349]	0.349
CDC light trap	1782	7.425 [7.088, 7.778]	0.871 [0.817, 0.930]	< 0.001
Ifakara tent trap – C	369	1.538 [1.388, 1.703]	0.180 [0.161, 0.202]	< 0.001
Window exit trap	54	0.225 [0.172, 0.294]	0.025 [0.019, 0.033]	< 0.001
Resting boxes indoor	6	0.025 [0.011, 0.056]	0.003 [0.001, 0.006]	< 0.001
Resting boxes outdoor	18	0.075 [0.047, 0.119]	0.008 [0.005, 0.013]	< 0.001

^a^ As described in the methods section, village, season and treatment were all included as fixed effects while household and date were included as random effects. In sampling *An. quadriannulatus,* both village and treatment did not significantly affect (*P* = 0.894 and 0.0845 respectively), the catches of mosquitoes by all methods. The catches of *An. funestus* were also significantly affected by village (*P* = 0.004) and treatment (p = 0.011). The catches of other anophelines and culicines were not significantly affected by village (*P* = 0.268 and 0.265) and treatment (*P* = 0.717 and 0.721) respectively. The catches of all the mosquito taxa were significantly affected by season (*P* < 0.001).

^b^ Mean and 95% confidence intervals (CI) were estimated by fitting generalised linear mixed models as described above, except that only method, date and house were included in a model without intercept.

^c^ Sensitivity of the sampling method catch with reference to HLC placed indoors (RR indicate Relative Rate).

^d^ Reference method.

^e^ Not applicable.

^f^ Not estimable due to no mosquito catch.

Statistical estimates of the magnitude and significance of differences in relative rates at which each trapping method captured mosquitoes are presented in Table 1. Of all the alternative methods, only CDC-LT performed better than HLC indoor for sampling both *An. quadriannulatus* and *An. funestus*, being over one and a half times more sensitive for both species. For *An. funestus*, ITT-C placed outdoors exhibited over three fourths the sensitivity of HLC and may therefore be useful for trapping this malaria vector species. However, for *An. quadriannulatus*, other anophelines and culicines, indoor CDC-LT proved the only reasonably sensitive alternative to HLC. For culicines, indoor CDC-LT exhibited more than three fourths the sensitivity of HLC which yielded approximately equal catches indoors and outdoors. While the ITT-C was the only alternative method other than CDC-LT that caught any useful numbers of culicines, it exhibited quite low sensitivity and might have limited utility for this important taxon that transmits a wide range of parasites and viruses of public health importance. ITT-C also exhibited extremely poor sensitivity for *An. quadriannulatus* and other anophelines. However, the RBs and the WET sampled much lower catches for all the mosquito taxa. Mosquitoes were observed on several occasions escaping from the RBs placed outdoors at sun rise prior to collection time.

### Influence of indoor residual spraying on the catches of *An. funestus* by all sampling methods

Supplementation of LLINs with IRS had no influence on the catches of *An. funestus* by indoor HLC (*P* = 0.270), outdoor HLC (*P* = 0.242) and CDC-LT (*P* = 0.229) placed indoors. While IRS appeared to increase catches in ITT-C placed outdoors (RR [95%CI] = 1.399 [1.016, 1.929], *P* = 0.040), this apparent effect is most likely spurious, arising from the relatively small number of houses assigned to each treatment.

### Influence of sampling method on the proportion of all fed *An. quadriannulatus* and *An. funestus* captured

All specimens of *An. quadriannulatus* caught with RBs placed outdoors had previously fed. HLCs, CDC-LT and ITT-C each collected less than a third of the fed *An. quadriannulatus* while RBs placed indoors and WETs caught none. However, RBs placed both indoors and outdoors collected high proportions of fed *An. funestus*. Approximately over a third of *An. funestus* mosquitoes caught by HLC and WET had fed. The former is a remarkably high proportion for a sample of host-seeking vectors and it is reassuring that this proportion is reduced in samples from both ITT-C and CDC-LT that are assumed to protect the human participant from exposure to the collected mosquitoes (Table 2).

**Table 2:** **Influence of sampling method on the proportion of fed *An. quadriannulatus *and *An. funestus *which were captured**^**a**^

**Sampling method**	**Percentage (Proportion fed)**	**OR**^**b **^**[95% C.I]**	***P *value**
*Anopheles quadriannulatus*			
HLC indoor	24.4 (99/405)	1.00^c^	NA^d^
HLC outdoor	29.3 (71/242)	1.900 [1.253, 2.881]	0.003
CDC light trap	12.9 (101/784)	0.417 [0.292, 0.596]	< 0.001
Ifakara tent trap – C	28.6 (6/21)	1.251 [0.430, 3.642]	0.682
Window exit traps	(0/1)	NE^e^	NE^e^
Resting boxes indoor	(0/0)	NE^e^	NE^e^
Resting boxes outdoor	100 (2/2)	NE^e^	NE^e^
*Anopheles funestus*			
HLC indoor	34.8 (608/1749)	1.00^c^	NA^d^
HLC outdoor	37.2 (608/1635)	1.188 [1.017, 1.387]	0.030
CDC light trap	20.6 (541/2630)	0.543 [0.467, 0.633]	< 0.001
Ifakara tent trap – C	14.1 (199/1410)	0.261 [0.215, 0.317]	< 0.001
Window exit trap	38.0 (27/71)	1.086 [0.643, 1.835]	0.758
Resting boxes indoor	73.7 (28/38)	4.486 [2.059, 9.776]	< 0.001
Resting boxes outdoor	72.7 (80/110)	5.899 [3.688, 9.434]	< 0.001

^a^ As described in the methods section, village, season and treatment were all included as fixed effects, while household and date were included as random effects. The proportions of fed *An. quadriannulatus* and *An. funestus* were not affected by village, season and treatment (*P* > 0.05).

^b^ Odds Ratio represents the relative probability of sampled mosquitoes which had fed compared to the reference indoor HLC method.

^c^ Reference method.

^d^ Not applicable.

^e^ Not estimable due to small or no numbers observed or no mosquito catches.

## Discussion

Amongst the methods that capture host-seeking mosquitoes, the CDC-LT placed near an occupied net compares well with HLC. This observation is consistent with many reports from elsewhere in the tropics in sampling various pathogen-carrying mosquito species [14,15,17,31,35,40,42,55-60] except for an evaluation in Dar es Salaam, Tanzania which showed very poor sensitivity of CDC-LT in this urban environment. While previous studies were limited to *An. gambiae s.s.* and *An. arabiensis* in Tanzania [40-42], this is the first report showing that ITT-C appears to be a useful option for sampling host-seeking *An. funestus* in an external trial site in Zambia. This species is among the most important malaria vectors in Africa generally and Zambia specifically, and it is notable that the ITT-C sampled considerably more *An. funestus* than any other mosquito taxon in this study. This is particularly noteworthy because ITT-C is the only sampling tool that has yet been successfully applied through quality assured community-based trapping schemes with epidemiological predictive power as a malaria risk indicator [43]. ITT-C might, therefore, be applicable as an option for programmatic use across much of Africa where *An. funestus* is an important vector of malaria [61,62]. Nevertheless, the poor sensitivity ITT-C exhibited for culicines, *An quadriannulatus* and other anophilines suggests caution, and that it requires evaluation across a broader diversity of contexts before it can be recommended for wide spread use. Indeed it has recently been emphasised that there is a great need to consistently compare sampling methods across diverse transmission patterns in Africa and that such comparative evaluations are conspicuous by their absence from the literature [1]. Critically, this study used a very similar design to that previously implemented in Dar es Salaam, so that the two evaluations from two very different contexts can be directly compared.

The observation by Govella *et al*. [40] that houses have many, highly variable entry and exit points, was also noted in our study area and might well explain the very low sensitivity of WET. The poor sensitivity of RBs is most likely explained by the fact that they represent too small a proportion of the total suitable resting surface area available to mosquitoes indoors and especially outdoors. Outdoor resting tools are also prone to natural mosquito predators which may contribute to the low catches [63] and mosquitoes also tend to leave when illumination increases as sunrise approaches. While other reports describe useful sensitivity levels of boxes [26,36] and pots [34] as resting traps, our observation that both the RB and WET methods exhibited poor sensitivities for sampling all mosquito taxa are consistent with some previous reports from neighbouring Tanzania [37,40]. Much of the dramatic drop in capture efficacy reported by these recent studies in Zambia (Table 1) and Tanzania [40], relative to previous reports from Kenya [26,34] and Tanzania [36] may well be explained by the presence and coverage levels of insecticidal nets. Given that insecticide-treated nets are estimated to prevent an average of 93% of exposure for people sleeping under them [48], it is inevitable that this study and a similar recent one in Tanzania in which all occupants used nets [40] both report far lower catches in resting traps than host-seeking traps because only a small minority of host-seeking mosquitoes will successfully survive, acquire a blood meal and consequently rest in the same house they entered.

However, this cannot entirely explain the comparatively low numbers of mosquitoes caught with resting boxes (≤ 1% sensitivity relative to HLC for all taxa except *An. funestus*). In the case of the WET, any deterred mosquitoes are readily available for capture upon exit, as demonstrated by recent trials of completely intact nets in experimental huts combining baffled entry points with comprehensive exit trapping of all remaining eaves and windows [64,65]. Fundamental limitations of sampling sensitivity of these RBs and WET formats are therefore probably important so more sensitive approaches such as PSC [5] and backpack aspirators [66] should be evaluated in a similarly standardised way. While these resting traps may be useful for some applications in some settings, the inferring quantitative levels of human exposure based on absolute numbers of mosquitoes caught may not be reliably recommended or readily interpreted in a standardized way. However, it is crucial to consider whether the focus of a given entomological survey is to quantify human exposure, understand vector resting behaviour, or identify blood meal sources of fed mosquitoes when selecting appropriate sampling tools [5]. Therefore sensitivity may not be the most important criterion in many cases.

In our study site, *An. quadriannulatus* appears to be the predominant species amongst the *An. gambiae* complex and was caught more indoors than outdoors by the CDC-LT and HLC methods. While these results seem unexpected because *An. quadriannulatus* is usually associated with outdoor biting and a preference for non-human hosts [31,50], it does occasionally bite people [67,68] but is thought to contribute negligibly to malaria transmission [67,68]. Torr and colleagues (2008) showed that, when humans are indoors, their odour attracts more zoophilic species than those stationed outdoors and this may partially explain the results obtained in this study [68]. The high numbers of *An. quadriannulatus* caught indoors here may also result from the fact that, apart from the catcher, other household inhabitants were present but covered with nets inside these homes, whereas the human single baits collected outdoors were alone. While the preference of *An. funestus* for feeding indoors was statistically significant (Table 1), it was quantitatively very small and of little biological significance, as appears to be the case for most malaria vector populations in Africa [69]. The vast majority of human exposure occurs indoors in this setting, and elsewhere in Africa [69] simply because the peak hours of *An. funestus* biting activity coincide with almost all humans going into their houses to sleep [63].

Although IRS treatment of houses which already had LLINs appeared to have no impact on the catches of *An. funestus* across all trap types, it appeared to increase catches by ITT-C placed outdoors. This presumably spurious result probably arises from the small number of houses assigned to each treatment because it is inconsistent with results reported here for the gold-standard HLC method and reported previously using logistic models of the proportion of mosquitoes caught indoors rather than outdoors at a given house [63]. So overall, it is notable that IRS with deltamethrin had so little apparent impact on house entry and subsequent host attack rates. This observation is consistent with a number of recent experimental hut evaluations [64,70,71] of modern pyrethroid formulations, confirming that this intervention format provides little direct protection to individual households and acts exclusively through community-level suppression of vector populations and malaria transmission.

It is worth noting that whilst large numbers of both *An. quadriannulatus* and *An. funestus s.l*. were caught indoors by the CDC-LT and HLC, the majority that had fed were sampled by the sampling methods placed outdoors. It is disconcerting that 24 to 37% of mosquitoes caught by the HLC methods, especially *An. funestus*, were blood fed. Presumably most of these either partially fed elsewhere before landing on the human bait to complete the blood meal, or obtained the blood meal from the human bait conducting the HLC. This supports the efforts to search for safer alternatives because these findings suggest that the catchers may have lacked concentration due to exhaustion and were therefore bitten extensively. High proportions of fed mosquitoes were also sampled in the RBs indoors and outdoors because these represent artificial resting places for mosquitoes, which rest most during the gestation phase of their life cycle. The lower proportions of fed *An. funestus* that were sampled by the ITT-C and fed *An. quadriannulatus* that were sampled by CDC-LT (Table 2) suggest that these methods do protect the human participants acting as bait and confirm the findings of Govella *et al*. in an urban Tanzanian setting [40]. It is possible that the substantial proportions of fed *An. funestus* and *An. quadriannulatus* in the ITT-C could have used the tent trap as an alternative resting place after feeding elsewhere or were simply attracted to the host for further feeding after being partially fed elsewhere. However, we could not substantiate this because our study did not include host blood meal analysis.

Despite these ambiguities and study limitations, these experiments do demonstrate the importance of evaluating the efficacy of alternative exposure-free sampling tools for routine monitoring of malaria transmission, in comparison with each other and with gold standard HLC in different settings. It further highlights the need to specifically evaluate sampling methods based on their ability to selectively trap either host-seeking, exiting, or resting mosquitoes, and to capture them with sufficient sensitivity relative to absolute house entry and host attack rates within houses.

## Conclusions

Although CDC-LT seems to be the most sensitive option for trapping host-seeking mosquitoes in this setting, the continuous need to recharge batteries might be challenging for surveillance systems in rural communities, particularly where electricity is not readily available. This may pose particular challenges for routine programmatic monitoring applications outside of research studies, notably community-based trapping schemes with little supervision and only occasional quality assurance [43]. The ITT-C appears to offer a reasonable alternative that does not depend on electrical power. However, its bulkiness could be a significant disadvantage that may limit its application in routine malaria surveillance systems, especially community-based schemes with little or no motorized transport. While RBs collect high proportions of fed mosquitoes, they have very low relative sensitivity in comparison with host-seeking methods, so similarly standardized evaluation of more promising methods for capturing resting mosquitoes, such as mechanized aspirators [66] and pyrethrum spray catch [5] should be considered. The efficacy of neither CDC-LT nor ITT-C appears to be affected by the application of pyrethroid-based IRS to houses already containing LLINs.

## Competing interests

Authors declare that they have no competing interests.

## Authors’ contributions

CHS, AS and GFK designed the study collected data analysed and drafted the manuscript. TLR: Assisted at the inception of the study through data entry and field design operations. JC and DC: Collected field data and morphologically identified all the mosquitoes in the field. JM: Provided the study map and assisted in the data analysis AS: Together with CHS and GFK designed the study and oversaw the field implementation of the study and contributed to the formulation of the hypothesis and drafting the manuscript. GFK: Together with AS and CHS designed the study and conceived the study hypotheses’ and contributed in drafting the manuscript. All authors read and approved the final version of the manuscript.
